# The Progress of Research on Genetic Factors of Recurrent Pregnancy Loss

**DOI:** 10.1155/2023/9164374

**Published:** 2023-03-24

**Authors:** Qinlan Li, Shuting Chen, Xinyi Dong, Sen Fu, Tianyu Zhang, Weiwei Zheng, Yonghong Tian, Donghui Huang

**Affiliations:** ^1^Institute of Reproduction Health Research, Tongji Medical College, Huazhong University of Science and Technology, Wuhan 430030, Hubei, China; ^2^NHC Key Laboratory of Male Reproduction and Genetics (Family Planning Research Institute of Guangdong Province), Guangzhou 510600, Guangdong, China; ^3^Department of Reproductive Endocrinology, Women's Hospital, School of Medicine, Zhejiang University, Hangzhou 310006, Zhejiang, China

## Abstract

Recurrent pregnancy loss (RPL) is both mental and physical health problem affecting about 1–5% of women of childbearing age. The etiology of RPL is complex, involving chromosomal abnormalities, autoimmune diseases, metabolic disorders, and endometrial dysfunction. The causes of abortion are still unknown in more than 50% of these cases. With the development of science and technology, an increasing number of scholars focus on this field and find that genetic factors may play an essential role in unexplained RPL, such as embolism-related genes, immune factor-related genes, and chromosomal numeric, and structural variation. This review summarizes the genetic factors associated with RPL, including genetic mutations and genetic polymorphisms, chromosomal variants, and chromosomal polymorphisms. Many related genetic factors have been found to be demographically and geographically relevant, some of which can be used for risk prediction or screening for the etiology of RPL. However, it is difficult to predict and prevent RPL due to uncertain pathogenesis and highly variable clinical presentation. Therefore, the genetic factors of RPL still need plentiful research to obtain a more accurate understanding of its pathogenesis and to provide more detection means for the screening and prevention of RPL.

## 1. Introduction

Recurrent pregnancy loss (RPL) is a common human reproductive disorder with an increasing incidence that affects approximately 1–5% of women of reproductive age [1]. It is estimated that the average prevalence of RPL for pregnant women is between 1–4% based on data from large-scale studies in Europe and the United States, in which approximately 50% of women suffer from unexplained RPL [2, 3]. The European Society of Human Reproduction and Embryology (ESHRE) defines RPL as three or more consecutive failed pregnancies at 20–24 weeks of gestation [4], and the American Society for Reproductive Medicine (ASRM) defines RPL as being two or more failed pregnancies [5]. The Royal College of Obstetricians and Gynaecologists (RCOG) defines RPL as fetal loss occurring three or more times consecutively with the same sexual partner and before the 24th week of gestation. RPL is multifactorial, and its pathogenesis involves multiple risk factors. These include abnormal uterine anatomy, genetic defects (parental chromosomal abnormalities), endocrine and metabolic disorders (hypothyroidism, diabetes mellitus), thrombosis, and autoimmunity (antiphospholipid syndrome) [6–8]. Although these and other associated factors have been identified, the exact cause of more than half of RPL etiologies remains unclear [9–11]. There are also many studies demonstrating the association of pregnancy loss with a woman's age, with the lowest risk of pregnancy loss in women aged 25–29 years (9.8%), increasing in women aged 30–35 years, and then rising sharply to 33.2% in women aged 40–44 years [12]. With the development of reproductive genetics, there have been many advances targeting genetic polymorphisms and mutations, karyotypic abnormalities, and embryonic chromosomal abnormalities in RPL couples, and the rate of embryonic chromosomal abnormalities was found to be 60% in the general population [13] and the incidence of RPL was 29%–60% [14–16]. Therefore, this article will review the abovementioned genetic factors of RPL.

## 2. Method

Criteria for selecting the subjects were as follows: Genetic factors associated with recurrent pregnancy loss. To access the literature: select PubMed as the search database and search with “recurrent pregnancy loss, genetic factors, genetic polymorphism, chromosomal abnormalities” as the keyword. There were many pathogenic factors related with RPL, such as gene polymorphism and mutations, karyotypic abnormalities, and embryonic chromosomal abnormalities. Many articles suggested polymorphisms in genes associated with RPL including angiogenesis, thrombogenesis, immune, and the estrogen receptor. A few suggested new possibilities are metalloproteinase gene polymorphisms, ATP 6V1G3 gene, cytoplasmic GST genes, and CLOCK gene. A number of articles clarified chromosomal aberrations associated with RPL including chromosome number abnormalities and chromosomal structure abnormalities (translocation, inversion, etc.). A small group of articles intimated new possibilities, such as closed placental chimerism and skewed *X* inactivation.

## 3. Mutations and Gene Polymorphisms

Gene polymorphism means that the structure or nucleotide arrangement of the same gene may vary between individuals. It is an allelic variation that does not necessarily affect the function of the gene but can be used as a marker to distinguish individuals. Its formation mechanism is a gene mutation.

### 3.1. Genes Associated with Angiogenesis

The generation of placental villi and embryonic vasculature is a critical step throughout embryonic development and is the foremost condition for embryo implantation. The major inducers of angiogenesis are essential for stimulating trophoblast proliferation, embryonic vascular development, and the growth of maternal and fetal blood cells during early pregnancy [17]. Vascular endothelial growth factor (VEGF) and nitric oxide synthase (NOS) are possible regulatory factors associated with RPL. VEGF gene polymorphisms affect protein expression by altering the transcriptional activity of the gene. Insufficient expression of VEGF affects the production of placental villi and metaplastic vessels, resulting in an inadequate blood supply to the embryo and causing impaired embryonic development, leading to RPL. NOS is a key enzyme in nitric oxide (NO) metabolism. Genetic polymorphisms can lead to conformational changes in endothelial nitric oxide synthase (eNOS) and affect eNOS activity, resulting in reduced NO synthesis. NO is a smooth muscle relaxant, and reduced NO synthesis leads to decreased vascular permeability and placental blood flow, thereby inhibiting embryo implantation. NO levels also regulate placental chorionic gonadotropin, which is associated with embryonic development [13].

As an angiogenic factor that may be associated with RPL in several populations [18], VEGF plays a significant role in fetal and placental angiogenesis. Moreover, placental VEGF is secreted from the endometrium, placenta, and endothelial and vascular smooth muscle cells [19]. The receptor-containing kinase insertion domain, also known as VGEF receptor 2, has been reported to have angiogenic effects on the placenta via the VGEF-KDR pathway [18, 20]. Several single nucleotide polymorphisms (SNPs) of the KDR gene have been reported to related to various diseases, such as nonsmall cell lung cancer, breast cancer, coronary heart disease, and RPL. However, the effect of KDR varies with different ethnic groups [21]. Many genetic association studies have examined the possible link between SNPs in VEGF and RPL susceptibility. For example, a recent meta-analysis [22] showed that polymorphisms in rs1570360, rs3025039, rs2010963, and rs3025020 were associated with RPL susceptibility. A later study [23] showed that the 1612G > A and 1725G > A polymorphisms in the VEGF 3′-UTR were relevant to RPL susceptibility in Korean women and that the VEGF 3′-UTR polymorphisms could be used as biomarkers for detecting RPL risk. The researchers also found increased expression of VEGF and its soluble Fms-like tyrosine kinase-1 (sFlt-1) during normal placental development, suggesting that VEGF signaling is a key hub for embryonic angiogenesis and vasculogenesis during placental development. One of the pathological features of RPL is dysfunctional angiogenesis and vasculogenesis, which implies that VEGF dysregulation may the relevance of RPL [24]. In addition to VEGF, it has also been shown that reduced Cx43 expression may also contribute to vascular dysfunction and angiogenesis disorders [25].

The G894T allelic variant of the NOS3 gene has a protective effect against the development of RPL in women. Consequently, the G894T allele variant may be a causal factor in the development of the disease [26]. However, more genetic association and functional studies in different populations are necessary to clarify the contribution of NOS3 + 894 G/T gene variants to IRSA [27]. Shin et al. [28] investigated three common polymorphisms of the eNOS gene (−786T > C, 4a4b, 894G > T) and RPL. eNOS 894GT + TT genotype and—786T—4b—894T haplotype were concluded to be significantly associated with RPL in Korean women. Furthermore, Parveen et al. [29] found that at least three common polymorphisms in the eNOS gene, namely, 12862A > G, Glu298Asp, and intron 4 VNTR, increased the risk of RPL in North Indian women. The abovementioned factor may elucidate that there are significant regional differences in VEGF and NOS gene polymorphisms, and more samples are needed to draw accurate conclusions.

Both VEGF and NOS have some population specificity, and mutations in their different loci may correlate with RPL in different regional populations, and this should be considered when determining the etiology of RPL.

### 3.2. Genes Associated with Thrombogenesis

The genetic polymorphisms associated with thrombogenesis are methylenetetrahydrofolate reductase (MTHFR) C677T, Factor V (FV) G1691A, Factor II (FII) G20210A, plasminogen activator inhibitor-1 (PAI-1) 5G/4G, etc. Mutations in these genes can cause persistent hypercoagulation and thrombotic tendency, leading to spontaneous abortion, but their correlation with RPL varies across geographic regions and populations [30, 31].

Among the mechanisms leading to RPL are as follows: (1) The increased frequency of mutated genes in the C667T locus of methylenetetrahydrofolate reductase (MTHFR) leads to a reduction in the action of MTHFR enzyme activity, causing high plasma homocysteine and low folate levels, which consequently brings about adverse pregnancy outcomes such as spontaneous abortion and abnormal embryonic development; (2) coagulation factor V (FV) active protein C (APC) controls the content and activity of coagulation factor V. Genetic polymorphisms cause APC resistance, which causes inactivation of coagulation factor V and increases blood hypercoagulation causing RRL; (3) during coagulation, mutations in the coagulation factor II (FII) gene lead to an increase in the amount of FII in the blood, which is converted from coagulation factor Va (FVa) to thrombin, leading to cause thrombosis; (4) mutations in fibrinogen activator inhibitor (PAI-1) occur and prevent fibrinolysis, leading to placental vascular thrombosis [13].

A related study reported the relationship between genetic polymorphisms of thrombogenic factors and RPL and found that FV G1691A and FII G20210A G/A heterozygous genotypes were high-risk factors for RPL occurrence, and PAI-1 5G/4G heterozygous genotype was a low-risk factor for RPL occurrence. In contrast, MTHFR C677T genotype was not directly related to RPL occurrence [32]. Later, it has also been shown that women with MTHFR 677TT (pure mutation, TT) genotype have markedly lower vitamin D levels, higher homocysteine, and natural killer (NK) cytotoxicity compared to women with MTHFR 677CC (wild type, CC) and 677CT (heterozygous mutation, CT) genotypes [33]. Fibrinogen activator inhibitor type 1 (PAI-1) regulates fibrinolysis, and the joint promoter region variants −675G/A (4G/5G) and −844G/A are associated with an increased risk of thrombosis. The association of PAI-1 variants with increased risk of RPL was also demonstrated by Magdoud et al. experiment [34].

One study [35] investigated 145 women with at least two consecutive miscarriages and 135 women with at least two children, and no history of miscarriage, genotypes of MTHFR C677T, and FVL and FII (prothrombin) polymorphisms were detected by real-time PCR. Information about exposure to environmental risk factors was also collected and no statistically diverse genotypes or allele frequencies were found for polymorphism studies, either in the women's RPL group or in the control group. Therefore, they concluded that such polymorphisms should not be considered risk factors for RPL in this population. Other studies have also reported no remarkable difference in the frequency of specific thrombosis-related mutations in women with a history of at least two miscarriages compared with women without pregnancy failure, which illuminates that obstetric failure may depend on the total number of individual mutations rather than the presence of individual genetic mutations [36].

In summary, conclusions regarding the association between thrombogenesis-related genes and RPL are not uniform and may be geographically correlated, with some studies suggesting that mutations or genetic polymorphisms in a subset of thrombogenesis-related genes are associated with RPL. Meanwhile, some prospective cohort studies have not found an association between thrombophilia and adverse pregnancy outcomes. Therefore, more relevant, multiregional studies are required.

### 3.3. Immune-Related Genes

Fetal genes are determined by both paternal and maternal lines. As a semigenetic transplantation process, pregnancy usually requires effective immune regulation to maintain immune homeostasis to avoid miscarriage due to rejection by the maternal immune system [37]. Thus, immune imbalance plays a material role in RPL. Inflammation may be associated with RPL, and some inflammation-related genes have been reported to be expressed abnormally in women with RPL. It has been shown that the rs910352T allele of the SERPINA4 gene is considerably relevant to RPL susceptibility, that the SERPINA4 rs20707777AA genotype is also associated with an increased risk of RPL, and that the SERPINA4 rs2070777AA genotype may increase the risk of RPL in a southern Chinese population [38]. It has also been shown that the distribution of genotypes and allele frequencies of FAU rs769440 differed vastly between RPL cases and healthy controls [39].

#### 3.3.1. B Cell-Related Genes

One study [40] showed a significant decrease in mRNA expression of B-cell-associated factors IL-10 and PD-L1 and increased expression of genes BLIMP1, IRF4 and XBP-1 in patients with RPL. An abnormal increase in PD-1/PD-L1 is detrimental to pregnancy and increases maternal immune rejection, leading to miscarriage [41]. The result [42, 43] of one study showed that the levels of IL-10-synthesizing B cells in the stimulated total B cell population isolated from the peripheral blood of RPL patients were markedly lower compared to those of normal pregnant women, unraveling that a decrease in the number of these cells may contribute to RPL. The decrease in the peripheral blood IL-10-synthesizing B cells may prompt RPL pathogenesis [44].

#### 3.3.2. NK Cell-Related Genes

Natural killer cells (NKs) are the most pivotal cells in fetal-maternal immune tolerance induced by the interaction of maternal killer cell immunoglobulin-like receptors (KIR) with fetal leukocyte antigens (HLA). IL-10 may negatively regulate the cytotoxicity of uterine NK (uNK) cells affecting pregnancy [45]. In RPL women, elevated levels of NK cells and increased NK cytotoxicity are relative to an increased T helper 1 immune response. It has been shown that the suppressor gene KIR3DL1 is a protective factor and the activator genes KIR2DS2 and KIR2DS3 are risk factors for RPL [46].

NK cells are related to the decidual immune microenvironment, where the meconium immune cells at the maternal-fetal interface are predominantly composed of NK cells, macrophages, T cells, and a few other cell types (e.g., dendritic cells, NKT cells, etc.) [47]. It is suggested that abnormalities in the metaplastic immune microenvironment may be involved in the pathogenesis of RPL [48].

NK cells are also pertinent to TLR3, a type I transmembrane protein consisting of 904 amino acids and composed of four parts, namely, an extracellular region containing 23 LRRs, N- and C-terminal cysteine-rich flanking regions, a transmembrane region, and a cytoplasmic tail region containing TIR. TLR3 recognizes “non-self” origin of nucleotide derivatives [49]. TLR3 activates NK cells, which participate in the maintenance of pregnancy tolerance by regulating fertilized egg implantation and uterine vascular alterations, probably through the association with poly (I-C), but excessive NK cell activity may lead to embryonic resorption and thus induce abortion [50].

#### 3.3.3. HLA-Related Genes

The embryo derives half of its genetic inheritance from the father and develops in the uterine environment, similar to a hemizygote. Thus, the fetus may be rejected by the maternal immune system, and one of the most essential immune factors is HLA-G. HLA-G is a nonclassical HLA class I antigen highly expressed on embryonic trophoblast cells in the meconium [51].

HLA expression in trophoblast cells has been shown to play an important role in maternal-fetal interface immune tolerance, with specific KIR in women with RPL and HLA ligands in couples causing susceptibility to RPL. One study found a prevalence of HLA-DQ2/DQ8 haplotype positivity in 51.58% of the women with RPL included in their trial, which is 1.5–2 times higher than the general population, which is in the range of 25%–40%, resulting in a higher prevalence of HLA-DQ2/DQ8 polymorphism and poorer pregnancy outcomes [52]. A report exploring the relationship between KIR2DL2 and its cognate ligand HLA-C1, found that a decrease in inhibitory KIR (inhKIR) ligands may be responsible for insufficient trophoblast inhibition by maternal uterine NK cells, resulting in RPL pathogenesis. Specific KIR and HLA-C genotyping may also be used to predict reproductive outcomes in women with RPL [53].

#### 3.3.4. Genetic Polymorphisms in Interleukin Genes

Many interleukin cytokines play a role in human conception [51]. Variations in genes alter the corresponding protein expression levels. SNPs in promoters are suspected to affect transcription factor binding, which may affect interleukin production and therefore be associated with RPL [54]. IL-1*β* (−511C/T) polymorphism leads to an increase in IL-1*β* production and the proportion of NK cells in the lymphocyte population [55, 56], producing a pro-inflammatory effect, which is elevated in women with RPL. IL-6 plays a role in trophoblast function [57], and IL-6 (−634) promoter mutations directly reduce IL-6 transcription and expression, and this nucleotide alteration also provides a potential for NF-1 transcription factor binding sites [58]. Variants in the IL-18 promoter region affect IL-18 transcription and translation, and IL-18 protein expression is lower in patients with RPL [59]. Interleukins and the corresponding immune cells work cooperatively to maintain the immune homeostasis of the mother and fetus; an imbalance of interleukin cytokines may lead to miscarriage [60]. The relationship between some interleukin gene polymorphisms and RPL is consistent in studies, such as IL-1*β* (−511C/T), IL-6 (−634C/G), IL-10 (−1082G/A, −819T/C), IL-18 (−137G/C) and IL-18 (−105G/A) [61]. However, in a small number of papers, interleukin genes have been linked to RPL, which may be influenced by factors such as race.

### 3.4. Genetic Polymorphisms in the Estrogen Receptor Gene

Estrogen is necessary for the maintenance of a successful pregnancy, and deficiency of estradiol in the luteal phase is associated with an increased risk of pregnancy loss [62]. Estrogen passively diffuses into the cell, where it binds to and activates its cytoplasmic receptor (ER), forming an estrogen-ER complex. This complex translocates to the nucleus, where it binds to specific DNA sequences of hormone response elements and regulates the transcription of target genes. There are two different ER forms ER*α* and ER*β*, with distinct tissue distribution and substrate specificity. ER*α* is encoded by the ESR1 gene located on chromosome 6 (6q25.1), whereas the ESR2 gene present encodes Er*β* on chromosome 14 (14q23.2) [63]. Recent studies have shown that genetic polymorphisms in ESR1 and ESR2 in linkage to RPL but these studies have no definitive results. Previous study found differences in estrogen and RPL in the Chinese population, and the AGT haplotype of the ESR2 gene with rs2077647A, rs4986938G and rs1256049T polymorphisms (ESR2 hapAGT) was a protective factor for URSA in Chinese Hui women [64].

Bahia et al. [65] conducted a study in which the main finding was the close association of the rs2234693 ESR1 gene variant with RPL. Their results are consistent with earlier studies from Germany [66] and Spain [67], but not with those from Brazil [68], Western Canada (Vancouver area) [69], Iran [70] and China [71]. This discrepancy is due to the different sample sizes between this and other studies [68], as well as differences in ethnic background [70, 71] and experimental setting [71]. They also investigated the possible connection of the rs3020314 ESR1 gene variant with RPL, but found no prominent linkage, which is inconsistent with an earlier German study that reported a negative correlation of the rs3020314 variant with the risk of RPL [72].

Accordingly, the association of estrogen receptor genes with RPL is also geographically specific and population-specific, and other relative studies are requisite.

### 3.5. Other Gene Polymorphisms

The genes mentioned below cannot be categorized into the gene types mentioned above, but during the literature search suggested a correlation with the development of RPL. Some of the genes have been confirmed by many experiments to be associated with RPL, while others are newly proposed by the investigators and may require more data for validation.

#### 3.5.1. Metalloproteinase Gene Polymorphisms

The regulation of matrix metalloproteinase proteins (MMPs) during embryo and placental implantation is pivotal for a successful pregnancy. In humans, 23 MMPs have been identified. MMPs are calcium-dependent zinc-containing endopeptidases that mediate ECM degradation, tissue remodeling, shedding of cell surface receptors, and processing of various signaling molecules [73]. A meta-analysis by Yan [74] showed that the MMP2 −735T allele and the MMP9 −1562T allele were closely integrated with the risk of RPL.

#### 3.5.2. ATP 6V1G3 Gene

The ATP 6V1G3 protein was predominantly expressed in the cytoplasm and stained brown. In the study of Chen [75], high expression of ATP 6V1G3 protein was found in placental villi and metaphase tissues, respectively. High expression of ATP 6V1G3 protein in women with RPL. However, its molecular mechanism in the development of RPL remains unclear.

#### 3.5.3. Genetic Polymorphisms of Cytoplasmic GST Genes

Oxidative stress (OS) [76] refers to the state of oxidative and antioxidant imbalance in the body. An essential prerequisite for normal metabolism, growth and development, is the provision of adequate oxygen during the embryonic, fetal and postnatal periods. The production of ROS due to hypoxia or hyperoxia, inflammation, or infection causes oxidative stress and changes in cell structure and function [77]. Defects in the maternal detoxification system may lead to RPL because the embryo is more exposed to exogenous and endogenous compounds. Many studies have shown that genetic polymorphisms in the cytoplasmic GST gene are associated with the risk of RPL [78–82]. It has been proposed that a genetic variant of the GSTA1 gene, the GSTA1-69C/T polymorphism (rs3957357), is significantly associated with the risk of RPL in Italian women with RPL [83]. However, some studies have also reported that the GSTA1-69C/T polymorphism is not significantly associated with the development of RPL in the Chinese Han Chinese population [84]. Therefore, the relationship between GST gene polymorphisms and RPL may also be related to the ethnic. In addition, sperm DNA is susceptible to oxidative damage, and increased sperm DNA fragmentation (SDF) may also lead to abnormal embryonic development [85].

#### 3.5.4. Genetic Variation in the CLOCK Gene

There is growing evidence that circadian rhythms affect a large number of physiological systems, including reproduction [86, 87]. Recent animal evidence unravels that disruption of synchronized clock activity relates to the pathogenesis of pregnancy complications. Repeated shifts in the light-dark cycle disrupt endogenous circadian rhythms and dramatically decrease the success rate of pregnancy in mice [88]. In addition, impaired reproductive capacity in humans has been closely linked with night work [89]. In humans, night shift workers have been shown to have increased rates of reproductive abnormalities and adverse pregnancy outcomes in terms of miscarriage, low birth weight and preterm birth [90]. Genetic variants in the circadian genes ARNTL and NPAS2 are thought to contribute to fertility, with genetic variants in the ARNTL gene being closely related to a higher number of miscarriages and specific genotypes of the Npas2 gene being associated with a reduced number of miscarriages [91]. Genetic variants in the circadian genes ARNTL2, CRY2, DEC1, PER3 and RORA have also been conjoined with an increased risk of premature placental abruption [92]. Additionally, it has been proved that low levels of CLOCK expression in pregnant women may lead to spontaneous abortion [93], and a study provided evidence that genetic variants in the CLOCK gene may be connected with IRSA [94].

#### 3.5.5. Mucin-Related Gene Polymorphisms

A recent study showed that MUC4 polymorphism correlates with RPL susceptibility in Korean women [95]. In this study, MUC4 rs882605 C > A and MUC4 rs1104760 A > G were strongly associated with an increased risk of RPL in Korean women. Mucin is secreted by the epithelial cells of the reproductive tissues to produce mucus of the cervix and endometrium, which plays an important role in reproductive processes [96]. Mucin 4 (MUC4) is the major mucin in the endometrial epithelium [96]. A study has found that MUC4 promotes cell migration, alters the endometrial environment, and creates weak spots in the epithelium, thereby prompting the failure of embryo implantation [96].

Thus, some genetic mutations and genetic polymorphisms are risk factors for RPL (Table 1), and it can be speculated that genetic mutations and genetic polymorphisms may occur in multiple concurrently, increasing the complexity of RPL etiology.

## 4. Chromosomal Abnormalities

### 4.1. Chromosomal Abnormalities in Embryos

Embryonic chromosomal abnormalities are a fundamental cause of RPL, primary infertility, mental retardation of the child, congenital malformations, growth retardation and other disorders. The incidence of embryonic chromosomal abnormalities in the general population is 60% [13], and the incidence of RPL is 29%–60% [14–16], most of which are chromosomal number abnormalities (96%), and a few are structural abnormalities (3%) [97].

#### 4.1.1. Chromosome Number Abnormalities in Embryos

Numerical abnormalities of chromosomes are classified as aneuploidy (trisomy, haploidy) and polyploidy, and chromosomal aneuploidy abnormalities are the most common, accounting for 70%, of which 60% are trisomic [97], followed by polyploidy and haploidy, 16-trisomy (12%–19%), 22-trisomy (4%–10%), and X-haploidy (6%–10%) are the most common [98]. Genetic risk factors for embryonic aneuploidy include meiotic errors, mitotic errors, and abnormal parental chromosome structure. Trisomies are usually the result of chromosome non-separation in maternal meiosis and commonly involve chromosomes 13, 16, 18, 21 and 22. At the same time, autosomal haploids are less common in monosomal abortions and are mostly X-sex chromosomes that occur as a result of the loss of the couple's *X* chromosome. Polyploidy, such as triploidy or tetraploidy, is usually caused by double spermatozoa or eggs that do not separate during maternal meiosis and are directly fertilized; tetraploidy may result from mitotic non-separation of the fertilized egg [99]. Maternal age was also found to be a primary risk factor for embryonic aneuploidy [100]; the proportion of aneuploid embryos increased from 25–35% in women under 35 years of age to 55–85% in women aged 40–45 years [101, 102].

#### 4.1.2. Embryonic Chromosomal Structure Abnormalities

Embryonic chromosomal abnormalities originate from two sources: first, chromosomal aberrations caused by internal and external factors during gamete formation or fertilized egg division; second, chromosomal abnormalities in either spouse that are inherited to the fetus, thus causing embryonic abortion or spontaneous miscarriage. Theoretically, embryos with unbalanced translocations cannot survive, while chromosomes with balanced translocations can survive with essentially preserved genetic material and no apparent abnormalities. However, most clinical studies have found that a few embryos with balanced translocation chromosomes can also miscarry, and other causes of miscarriage cannot be excluded [103].

### 4.2. Chromosomal Abnormalities in Couples

Chromosomal abnormalities are present in at least one partner in 3%–8% of RPL couples, 92.9% of which are structural abnormalities and a small amount of which are numerical abnormalities. Common chromosomal number abnormalities include Turner syndrome (45, XO), Klinefelter syndrome (47, XXY), superfeminine syndrome (triple *X* syndrome, 47, XXX) and double Y syndrome (47, XYY) [98]. Chromosomal structural abnormalities are dominated by translocations (including reciprocal balanced translocations and Robertsonian translocations), and in approximately 3.5% of couples, the parents are carriers of structural chromosomal rearrangements [104]. Others include chimerism, ring chromosomes, chromosomal insertions, inversions, duplications and deletions [12]. Parental chromosomal translocations, inversions and copy number variants are more common in couples with RPL (2–5%) than in the general population (0.7%) [104–107]. In couples with RPL, the male partner has 2.7 times the average rate of sex chromosome aneuploidy and 3–6 times the rate of aneuploidy on chromosomes 13, 18 or 21 [108].

#### 4.2.1. Translocation

**Figure 1:**
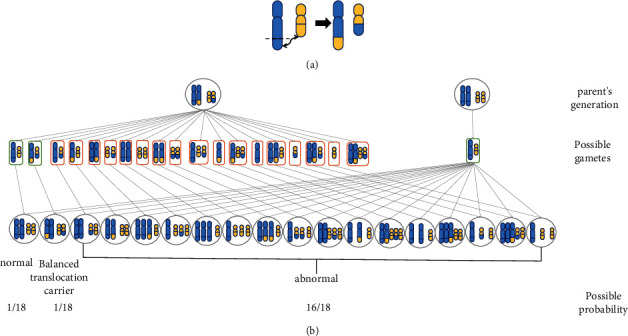
(a) Balanced translocations on nonhomologous chromosomes. (b) Possible gametes in patients with balanced translocations. During meiosis I, the translocated chromosome combines with its normal homologous chromosome to form a tetrad. Balanced gametes containing normal non-homologous chromosomes or two translocated chromosomes resulting from alternate segregation are designated by green border, and unbalanced gametes by red border. Chromosome segregation patterns for tetrad are shown: 2 : 2 (two non-homologous or two homologous chromosomes segregate together in an adjacent-1 or adjacent-2 segregation, respectively), 3 : 1 (three chromosomes segregate into one cell and one into the other), and 4 : 0 (all chromosomes segregate together).

Reciprocal balanced translocation (RBT) is formed by a mechanism in which two chromosomes break simultaneously and the broken fragments are exchanged to form two derived chromosomes, generally without increasing or decreasing in genetic material. Thus, the individual usually has no phenotypic alterations. Reciprocal balancing translocations (RBT) can occur between homologous or non-homologous chromosomes. Still, balancing translocations between homologous chromosomes cannot produce gametes, so we will only discuss the case of balancing translocations arising between non-homologous chromosomes. (Figure 1(a)). It has been reported that 18 gametes can be produced during gamete formation, only one of which is normal, and the rest are unbalanced gametes. Segregation was performed by five possible modes: alternate, adjacent-1, adjacent-2, 3 : 1 or 4 : 0 (Figure 1(b)). Alternating segregation produces only balanced gametes. Adjacent-1, Adjacent-2, 3 : 1 and 4 : 0 segregation will produce unbalanced gametes. Reciprocal balanced translocations occur in 0.195% of the general population, and the frequency of translocations is about 1.3% in infertile males [109]. In 3% to 6% of RPL, one of the two parents carries a chromosomal balanced translocation [37]. When an abnormal gamete binds to a normal egg or sperm, an imbalance in genetic material can induce monosomies or trisomies. Thus resulting in miscarriage and stillbirth.

**Figure 2:**
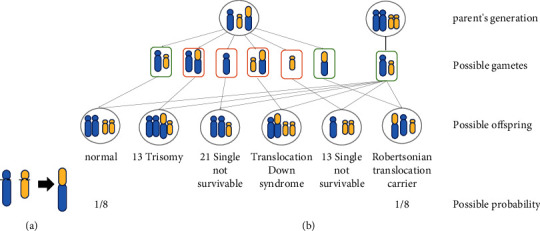
(a) Robertsonian translocations on non-homologous chromosomes. (b) Possible gametes in patients with robertsonian translocations. Non-homozygous robertsonian translocations can produce six types of gametes in the formation of germ cells, one normal, one balanced, and the remaining four unbalanced gametes. Normal and balanced gamets are designated by green border, and unbalanced gametes are designated by red border. The probability of normal and robertsonian translocation carrier are both 1/8.

Robertsonian translocation occurs in acrocentric chromosome and refers to the process in which two proximal chromosomes break at the trophectodomain to form a long-arm chromosome. Robertsonian translocations can occur between homologous or non-homologous chromosomes, but Robertsonian translocations between homologous chromosomes also fail to produce gametes. Therefore, we shall only summarize the case of non-homologous chromosome equilibrium translocations (Figure 2(a)). It is a specific form of translocation with an incidence of 0.1% in the general population. After translocation, the two long arms fuse with each other to form a larger chromosome, while the two short arms are often lost. The chromosomes in which translocations occur are classified as homozygous Robertsonian translocations or non-homozygous Robertsonian translocations. Non-homologous Robertson translocations can produce six types of gametes when forming germ cells, one normal, one balanced and the other four unbalanced (Figure 2(b)). Unbalanced gametes can cause abortions, malformations and stillbirths due to an imbalance of genetic material. In the case of homozygous Robertsonian translocations, the general offspring only have the possibility of forming translocated trisomies or monosomies.

Balanced translocations and inversions do not affect the phenotype of the parents themselves, but their unbalanced gametes during meiosis may indeed be partially responsible for abortion. Likewise, Robertsonian translocations of parental chromosomes may cause miscarriages, congenital disabilities or mental retardation in the offspring [110]. Chromosomes 11, 6, 4, 1 and 18 are the most common translocated chromosomes [111].

#### 4.2.2. Inversion

**Figure 3:**
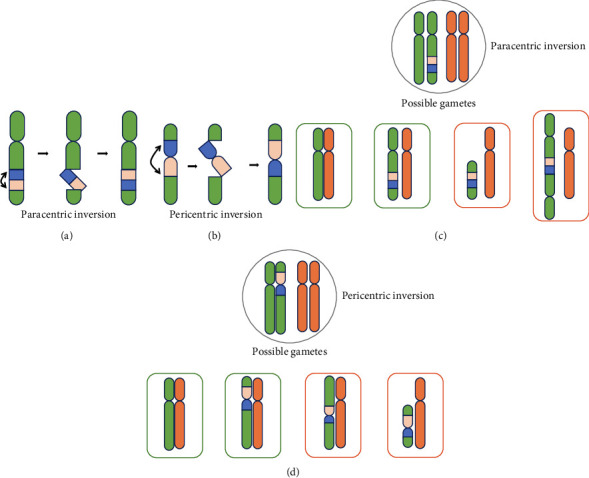
Chromosomal inversions: two breaks in the same chromosome, causing the resulting fragments to reconnect after 180 degrees of reversal. (a) Paracentric inversion: the inverted segments do not contain chromosomes. (b) Pericentric inversion: the inverted segments contain chromosomes. (c) Possible gametes of paracentric inversion. In meiosis, a crossover between a normal chromosome and an inverted chromosome results in the loss or duplication of a segment of the gametophyte chromosome, leading to chromosome abnormality and abnormal traits in the offspring. Balanced gametes are designated by the green border. Unbalanced gametes are designated by the red border. (d) Possible gametes of pericentric inversion. Balanced gametes are designated by the green border. Unbalanced gametes are designated by the red border.

An inversion is a rearranged chromosome formed when a chromosome breaks in 2 places, forming 3 segments. The middle segment is inverted by 180° and then joined to form a rearranged chromosome, which is divided into inter-arm inversion and intra-arm inversion (Figures 3(a) and 3(b)). Inverted chromosomes form an inversion loop during meiosis, and homologous chromosomes undergo recombination to produce four types of gametes, one normal, one inversion carrier, and the other two unbalanced gametes with partial duplication and partial deletion of no or double mitosis (Figures 3(c) and 3(d)), which, when combined with normal gametes, cause an imbalance of genetic material, resulting in abortion or stillbirth. Interarm inversions are most common on chromosomes 1, 9 and 11, with a prevalence of 1.0% in the population and 2.28% in RPL patients, observably higher than in the general domestic population. There are some controversies regarding the effect of the inversion of chromosome 9 on RPL. Some studies have illustrated that Inv (9) is the least common polymorphic variant in infertile couples [112], while Jeong et al. [113] also suggested that inter-arm inversions of chromosome 9 are normal variants and generally do not affect individual health. Most scholars believe that interarm inversion of chromosome 9 is a polymorphism and that carriers do not have an abnormal phenotype. However, an increasing number of studies clarify that it is closely related to abnormal clinical conditions such as infertility and RPL.

#### 4.2.3. Duplicates and Deletions

Chromosomal deletions and additions, called copy number variants (CNV) [105], are classified as large CNV (≥10 Mb) and submicroscopic CNV (<10 Mb). Nucleotide microarray technology was used to detect chromosomes in recurrent flow products, and small deletions of chromosome *X* were found in up to 6% of RPL women. Chromosome 16 duplications were the most common, followed by *X* chromosome deletions and triplet chromosome abnormalities, and again by chromosome 21 and 22 duplications. Minor deletion duplications of chromosomes, such as chromosome 2, 4, 9, 13, 14, 15, 17, 18 and 20 duplications were also found [112]. Larger deletions and increases in CNVs involving online human genetics (OMIM) genes and CNVs not found in large databases of normal individuals are likely to be associated with pregnancy loss, and pathological smaller CNVs (<400 kb) are of uncertain significance and may not be closely linked with pregnancy loss [105].

### 4.3. Chromosomal Polymorphism

Chromosomal polymorphisms are minor variations in chromosomes that can exist in normal populations, mainly in the size, morphology, and coloration of homologous chromosomes, such as variation in satellite of the D-G group, growth or shortening of chromosomal subconstrictions, and minor variations in the length of the Y chromosome. While chromosomal polymorphisms were previously thought to be non-pathological variants occurring in heterochromatin regions of chromosomes, including small variations in the structure, coloration intensity, and bandwidth, an increasing number of studies have shown that chromosomal polymorphisms increase the risk of developing RPL and are also associated with infertility, decreased sperm quality, and congenital disabilities. The mechanism of the clinical effect is that the variation in the heterochromatin region of chromosomal polymorphism affects the function of mitotic granules, as well as sister chromatid binding and chromosome segregation, adding to difficulties in homologous chromosome pairing, which affects cell division and thus causes embryonic developmental disorders, triggering the development of RPL. On account of chromosomal polymorphisms are also present in the normal population, it was previously thought that chromosomal polymorphisms were not the cause of RPL, but in recent years, several studies have shown a correlation between chromosomal polymorphisms and the occurrence of RPL.

The occurrence of chromosomal polymorphisms in the population should be relatively equal and stable. Meanwhile, the results of one study showed that chromosomal polymorphisms were more frequent in patients with RPL than in control patients, and the difference was conspicuous. In that study, it was also found that chromosomal polymorphisms frequently occurred in Chinese patients with RPL, implying that RPL in Chinese patients may be affiliated with chromosomal polymorphisms [52]. It has also been shown that 9 qh + polymorphism is the most observed variant in patients with recurrent miscarriage (RM) [113]. Amiel et al. [114] reported that the husband's inv (9) could increase the frequency of heterozygosity in sperm cells, which may lead to miscarriage in his wife and Down syndrome in the fetus.

### 4.4. Special Chromosomal Abnormalities

#### 4.4.1. Closed Placental Chimerism (CPM)

Restrictive placental chimerism occurs when all or part of the genetic makeup of the placenta differs from that of the fetus. Genetically abnormal placentas inextricably linked to placental insufficiency, fetal growth restriction and death [105]. Fetal growth restriction (FGR) was reported in 71.7% of CPM cases, and preterm birth (<37 weeks) was reported in 31.0% of cases. A high percentage of structural fetal malformations of 24.2% was also found in cases of CPM.

#### 4.4.2. Skewed *X* Inactivation

In females, partial or complete inactivation of one *X* chromosome in a particular cell during the embryonic period is called *X* chromosome inactivation [105]. The *X* chromosome inactivation (XCI) process begins at the preimplantation stage of human embryonic development, probably around the eight-cell stage [115]. The extreme skew of XCI (when defined as greater than 90%, the incidence of XCI is significantly higher) is associated with RPL. The essentiality of RPL is diminished when it is defined as two or more losses [116]. In Korea, skewed *X* chromosomes were not bound up with patients with RPL of unknown cause [115]. In a case-control study, curved XCI and shortened telomere length were found to be closely tied with idiopathic premature ovarian failure (POI) despite the absence of alterations in the androgen (AR) and FMR1 genes. Additionally, women with shorter telomeres tended to exhibit a skewed XCI [117]. In a study by Sharp et al. [118], the incidence of severe skewing was higher in women with idiopathic premature ovarian failure and increased with age, with an incidence of 7% in women younger than 25 years and 16% in women older than 60. Through Mark's research [119], solid statistical evidence was provided that female carriers of X-linked recessive fetal lethal defects are at incremental risk of RPL.

## 5. Conclusion and Future Directions

In summary, the etiology of RPL is complex and often results from a combination of multilinked abnormalities, with genetic factors involving not only abnormal karyotypes but also chromosomal polymorphisms (Table 2) and genetic abnormalities. However, due to differences in study sample size, geography, race, and population, many factors have not yet been uniformly concluded, and studies with expanded samples and increased geography are needed. Simultaneously, we should consider good genetic counseling and pregnancy screening in RPL prediction to detect problems early. In clinical practice, physicians should take a detailed medical history, and some ancillary tests are necessary to help screen for etiology. Patients with RPL should be monitored more closely during pregnancy, and if necessary, pregnancy should be terminated when appropriate.

## Figures and Tables

**Table 1:** Summarize the possible factors affecting RPL in gene mutations and gene polymorphisms.

Genes with different functions	Type	Functions	Genes/gene polymorphisms associated with RPL	Reference
Angiogenesis-related genes	VEGF	Stimulation of trophoblast proliferation, development of embryonic vasculature	Polymorphisms of rs1570360, rs3025039, rs2010963 and rs3025020	[22]
			The 1612G > A and 1725G > A polymorphisms in the VEGF 3′-UTR	[23]
	NOS	Increases vascular permeability	894G/T	[26, 27]
			−786T > C	[28]
			12862A > G; Glu298Asp; Intron 4 VNTR	[29]

Genes related to thrombosis	MTHFR	Maintenance of low plasma homocysteine levels	C677TT	[30, 31, 33]
	F V	Blood clotting	G1691A	[30, 31, 33]
	FII	Blood clotting	G20210A G/A	[30, 31, 33]
	PAI-1	Fibrinolysis	−675G/A (4G/5G);−844G/A	[30, 31, 34]

Immune-related genes	PD-1/PD-L1	T-cell immune response and immune homeostasis	PD-1/PDL-1 abnormal increase	[38–43]
	NKs	Induced immune tolerance in fetal mothers	KIR3DL1; KIR2DS2; KIR2DS3	[44, 45, 48]
	HLA	Induced immune tolerance in fetal mothers	HLA-DQ2/DQ8Polymorphism	[49, 50, 52]

Gene polymorphism of estrogen receptor gene	ESR1	Maintaining a successful pregnancy	rs2234693	[60, 63]
			rs3020314	[60, 70]
	ESR2	Maintaining a successful pregnancy	rs2077647A; rs4986938G; rs1256049T	[60, 61]

Inflammation-associated gene polymorphisms	SERPINA4	The body responds to injury or infection	rs20707777AA; rs910352T	[71]
	FAU	The body responds to injury or infection	rs769440	[72]

MMPs	MMP2	Helps implantation and stabilizes the placenta	MMP2-735T	[73–77]
	MMP9	Helps implantation and stabilizes the placenta	MMP9-1562T	[73–77]

ATPase-related genes		ATP synthesis, substance transport	ATP 6V1G3	[78]
Genes associated with oxidative stress	GST	Prevents oxidative damage	GSTA1-69C/T polymorphism	[80, 86, 87]

Genes associated with rhythm		Maintaining normal physiological functions of the body	ARNTL; Npas2	[94, 96, 97]
			ARNTL2; CRY2; DEC1; PER3; RORA	[95–97]

**Table 2:** Summarize the possible factors affecting RPL in chromosomal variation.

Types of chromosomal variants		Type	Possible risk factors	Commonly occurring abnormal chromosomes	Reference
Chromosomal abnormalities in embryos	Number anomalies	Aneuploidy (trisomy, monosomy)	Meiotic error, mitotic error and abnormal parental chromosome structure	Chromosomes 13, 16, 18, 21 and 22; x chromosome	[98, 101]
		Polyploid	Double sperm into the egg or meiotic non-separation of the egg; Mitotic non-separation of fertilized eggs		[100]
	Structural anomalies	Abnormal equilibrium translocation structure	Spontaneous mutation by internal and external environmental influences, inherited by couples carrying abnormal chromosome structure		[120]
		Non-equilibrium translocation structural abnormalities			

Chromosomal abnormalities in couples	Structural anomalies	Reciprocal balanced translocations	An exchange of DNA segments between nonhomologous chromosomes with no gain or loss of DNA	Chromosomes 11, 6, 4, 1, and 18 were the most commonly translocated chromosomes	[37, 111, 112]
		Robertsonian translocations			
		Inversion	Production of unbalanced gametes	Chromosomes 1, 9 and 11 are the most common	[113, 121]
		CNV	Gene deletion or increase	Chromosome 6 duplication was the most common, followed by *X* chromosome deletion and triplet chromosome abnormalities	[106, 122]

Chromosome polymorphism			Affects mitophase function, sister chromatid binding and chromosome segregation	Inv (9)	[104, 114, 115]

Special chromosomal anomalies		CPM	Placental insufficiency, fetal growth restriction and death		[106]
		XCI	Increased risk of spontaneous abortion in female carriers of X-linked recessive fetal lethal defects	x chromosome	[117, 118, 123], [124]

## Data Availability

No datasets were generated or analyzed during the writing of this review.
